# Production, Purification and Characterization of Recombinant, Full-Length Human Claudin-1

**DOI:** 10.1371/journal.pone.0064517

**Published:** 2013-05-21

**Authors:** Nicklas Bonander, Mohammed Jamshad, Dominik Oberthür, Michelle Clare, James Barwell, Ke Hu, Michelle J. Farquhar, Zania Stamataki, Helen J. Harris, Karsten Dierks, Timothy R. Dafforn, Christian Betzel, Jane A. McKeating, Roslyn M. Bill

**Affiliations:** 1 School of Life and Health Sciences, Aston University, Aston Triangle, Birmingham, United Kingdom; 2 Department of Chemical and Biological Engineering, Chalmers University of Technology, Gothenburg, Sweden; 3 Institute of Biochemistry and Molecular Biology, Laboratory for Structural Biology of Infection and Inflammation, University of Hamburg, Hamburg, Germany; 4 Center for Free-Electron Laser Science, Deutsches Elektronen-Synchrotron (DESY), Hamburg, Germany; 5 Institute of Biomedical Research and NIHR Center for Liver Disease, University of Birmingham, Birmingham, United Kingdom; 6 School of Biosciences, University of Birmingham, Birmingham, United Kingdom; National Institute for Viral Disease Control and Prevention, CDC, China

## Abstract

The transmembrane domain proteins of the claudin superfamily are the major structural components of cellular tight junctions. One family member, claudin-1, also associates with tetraspanin CD81 as part of a receptor complex that is essential for hepatitis C virus (HCV) infection of the liver. To understand the molecular basis of claudin-1/CD81 association we previously produced and purified milligram quantities of functional, full-length CD81, which binds a soluble form of HCV E2 glycoprotein (sE2). Here we report the production, purification and characterization of claudin-1. Both yeast membrane-bound and detergent-extracted, purified claudin-1 were antigenic and recognized by specific antibodies. Analytical ultracentrifugation demonstrated that extraction with n-octyl-β-d-glucopyranoside yielded monodispersed, dimeric pools of claudin-1 while extraction with profoldin-8 or n-decylphosphocholine yielded a dynamic mixture of claudin-1 oligomers. Neither form bound sE2 in line with literature expectations, while further functional analysis was hampered by the finding that incorporation of claudin-1 into proteoliposomes rendered them intractable to study. Dynamic light scattering demonstrated that claudin-1 oligomers associate with CD81 *in vitro* in a defined molar ratio of 1∶2 and that complex formation was enhanced by the presence of cholesteryl hemisuccinate. Attempts to assay the complex biologically were limited by our finding that claudin-1 affects the properties of proteoliposomes. We conclude that recombinant, correctly-folded, full-length claudin-1 can be produced in yeast membranes, that it can be extracted in different oligomeric forms that do not bind sE2 and that a dynamic preparation can form a specific complex with CD81 *in vitro* in the absence of any other cellular components. These findings pave the way for the structural characterization of claudin-1 alone and in complex with CD81.

## Introduction

Hepatitis C virus (HCV) is a member of the *Flaviviridae* family. This important human pathogen specifically infects the liver. At present there is no HCV vaccine and although a number of drugs targeting HCV replicase enzymes are in development, recent trials have shown a rapid appearance of drug-resistant viruses [1], [2]. The conserved nature of HCV entry into host cells offers an alternative and attractive target for therapeutic intervention.

HCV initiates infection by attaching to the cell surface followed by clathrin-dependent internalization of virus particles; current evidence supports a role for scavenger receptor class B member I (SR-BI), tetraspanin CD81 and tight junction proteins claudin-1 and occludin in coordinating this process (reviewed in [3]). SR-BI and CD81 bind HCV-encoded E1E2 glycoproteins with high affinity and have been reported to play a role in particle attachment to the cell [4], [5]. In contrast, there is limited information on whether claudin-1 or occludin interacts directly with HCV. The essential role of claudin-1 in the late stages of HCV entry [6] suggests that there may be a requirement for the virus to bind receptor proteins in a defined sequence or that claudin-1 has another, as yet undetermined, function.

**Figure 1:**
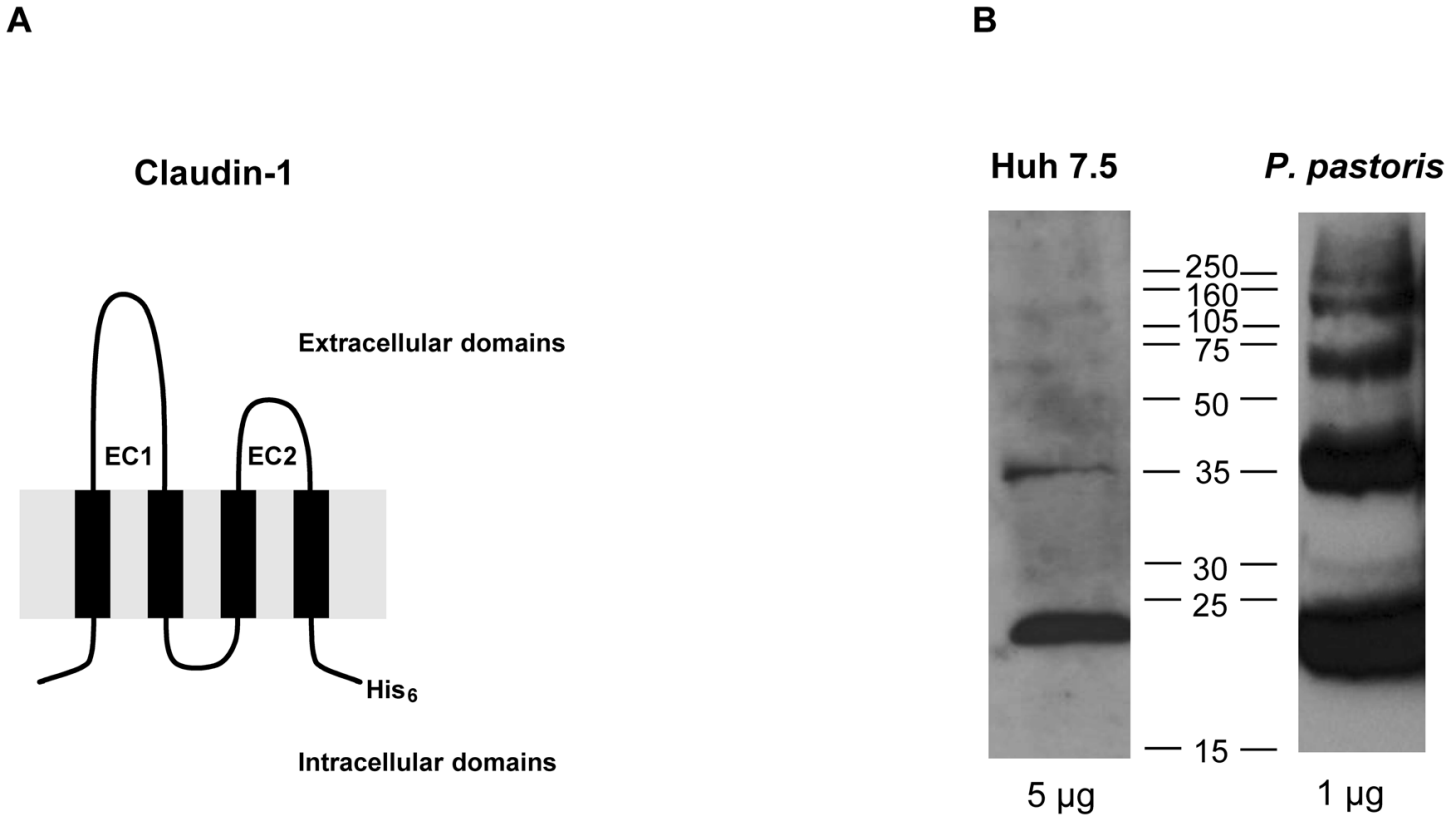
Claudin-1 forms monomers and higher order structures in yeast membranes. (A) A schematic representation of the claudin-1 protein produced in this study. The membrane is represented in grey (the extra- and intracellular sides are labeled), the extracellular loops, EC1 and EC2, are marked and the His_6_ tag is indicated in the carboxy-terminal tail. The predicted molecular mass is 23.7 kDa. (B) Recombinant claudin-1 in *P. pastoris* membranes forms monomers, dimers and trimers as determined by non-reducing SDS-PAGE (1 µg protein loaded per well). Endogenous claudin-1 in Huh-7.5 hepatoma cells was analyzed for comparison (5 µg protein loaded per well) showing monomers and oligomers. Protein concentrations in the two cell types are different because the yeast cells overexpress recombinant claudin-1; this does not affect the antigenicity of the protein, as shown in Fig. 3. Molecular size markers are indicated in the intervening lane.

The claudin superfamily of four transmembrane domain (4TM) proteins oligomerize to form strands that comprise cellular tight junctions [7], thereby generating the seal required to maintain cellular homeostasis. Interactions between the first and second claudin extracellular loops (EC1 and EC2; **Fig. 1A**) allow protein associations both within the plasma membrane of a single cell and between adjacent cells (reviewed in [8]); Förster resonance energy transfer (FRET) between tagged molecules suggests that protein dimers are the primary building block(s) of claudin strands [8]. We [9], [10], [11] and others [12] have reported that claudin-1 associates with tetraspanin CD81; this receptor complex is present at the basolateral membrane of hepatoma cells [13] and is essential for HCV entry *in vitro* [10]. Inhibiting protein kinase A [14] or activating epidermal growth factor accessory protein [15] limits claudin-1/CD81 complex formation and HCV entry. Furthermore, anti-claudin-1 antibodies inhibit HCV infection by reducing claudin-1 association with CD81 without perturbing tight junction integrity [16].

Having established the biological relevance of claudin-1 interaction with CD81, our aim is to characterize these proteins *in vitro*. Since primary human hepatocytes and the majority of human hepatoma cells express all four receptor proteins, and siRNA silencing approaches are frequently partial, it is difficult to study the role of claudin-1 in the HCV internalisation process in mammalian cells. We have therefore focused on purifying recombinant, full-length protein components of the HCV receptor complex; studies on CD81 have highlighted important differences in the interaction of HCV with full-length, cell-expressed forms of CD81 and recombinant truncated forms of the soluble second extracellular loop (EC2) [17]. For example a soluble, mutant form of EC2 (F150S) does not interact with HCV sE2, while mutation of the same residue in full-length, cell-expressed CD81 has minimal effects on HCV sE2 binding [18]. These results suggest that EC2 has a more robust structure in the full-length tetraspanin and, together with other data, indicate that regions in EC1 or the transmembrane domains may play a role in CD81 dimerisation [19].

We previously reported the purification of recombinant, full-length human CD81 from the methylotrophic yeast *Pichia pastoris* [20]: monomers, dimers and higher oligomers of CD81 were observed in recombinant *P. pastoris* membranes comparable with endogenous protein in mammalian membranes. Immunofluorescent and flow cytometric staining of *P. pastoris* protoplasts with monoclonal antibodies specific for CD81 EC2 demonstrated comparable conformation of the recombinant and native molecules. Recombinant CD81 isolated in a monodispersed form using n-octyl-β-d-glucopyranoside (βOG), as determined by analytical ultracentrifugation (AUC), was shown to interact with HCV sE2, representing the first biophysical characterization of a functional, full-length, recombinant tetraspanin [20].

Here we report the production of milligram quantities of recombinant human claudin-1 using the yeast, *P. pastoris*. Yeast protoplasts expressing claudin-1 bound an antibody specific for a conformation-dependent epitope expressed on human hepatocytes, suggesting a native protein conformation. Claudin-1 could be isolated from yeast membranes in various oligomeric forms, dependent upon the detergent used. When isolated with βOG, it was monodispersed and dimeric, while isolation with profoldin-8 or n-decylphosphocholine (foscholine-10) resulted in dynamic mixtures of oligomers. When oligomeric preparations were reconstituted into liposomes, the resulting proteoliposomes inhibited HCV infection of hepatoma cells in a dose-dependent manner. Claudin-1-containing proteoliposomes also inhibited the infectivity of lentiviral pseudotypes expressing HCV E1E2 glycoproteins (HCVpp) and, unexpectedly, vesicular stomatitis virus expressing glycoprotein G (VSV-Gpp). In contrast, CD82-containing proteoliposomes had no effect on HCVpp or VSV-Gpp, indicating that proteoliposomes are not tractable to study the biological function of claudin-1. In the absence of a suitable biological assay, the known interaction of claudin-1 with CD81 was therefore examined *in vitro*.

Using dynamic light scattering (DLS), dynamic preparations of claudin-1 (in foscholine-10) were demonstrated to associate with CD81 in a defined molar ratio (1∶2) of claudin-1∶CD81 and in the absence of any other cellular components. In contrast, monodispersed claudin-1 (in βOG) failed to associate with CD81. Claudin-1/CD81 complexes were stabilized by the presence of cholesteryl hemisuccinate (CHEMS) consistent with literature reports that cholesterol promotes complex formation in mammalian cells [3]. In summary, this study represents the first in-depth characterization of recombinant, full-length claudin-1; the data presented here should accelerate the structural analysis of the claudin superfamily and promote structure-aided design of therapeutic agents that target the early entry step of the HCV lifecycle.

## Results

### Full-length human claudin-1 is oligomeric in yeast membranes

Claudin-1 was expressed in *P. pastoris* cells using our previously reported protocol for CD81 [20]. SDS-PAGE analysis of human claudin-1 expressed in yeast membranes showed diverse oligomeric states, as previously reported for CD81 [20]. The oligomeric pools of claudin-1 observed in yeast membranes were comparable with endogenous claudin-1 observed in Huh-7.5 hepatoma cells under non-reducing conditions (**Fig. 1B**).

**Figure 2:**
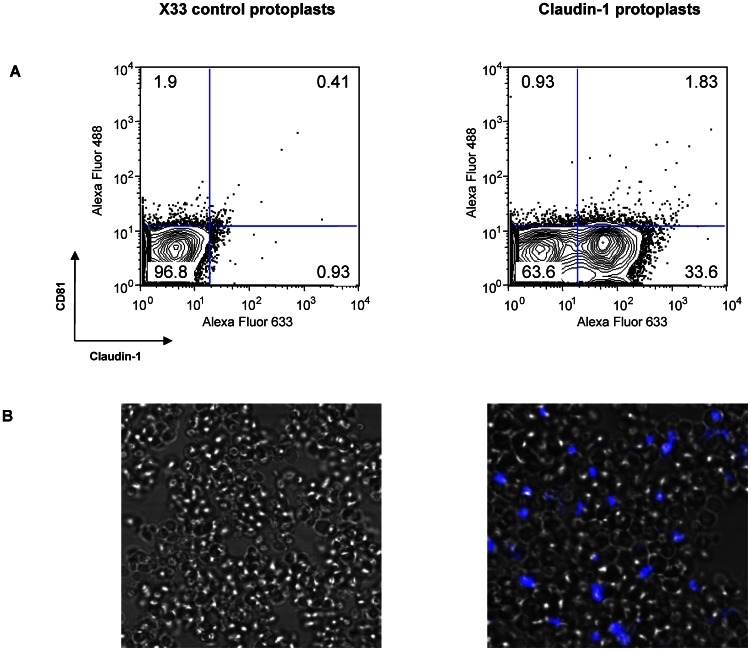
Antigenicity of human claudin-1 in yeast protoplasts. Conformation-dependent antibodies specific for claudin-1 (R&D Systems) and, as a control, CD81 (2s131) were used as tools to probe the antigenicity of yeast-expressed claudin-1. Secondary antibodies were Alexa Fluor 488 goat anti-mouse IgG (H+L) and Alexa Fluor 633 goat anti-rat IgG (H+L) (Invitrogen). The parental X33 strain with no heterologous protein expression was used as a negative control. (A) Fluorescence activated cell sorting shows specific antibody binding with anti-claudin-1 antibodies for X33 (0.93%) and claudin-1 (33.6%) protoplasts. For anti-CD81 antibodies, the corresponding values are X33 (1.9%) and claudin-1 (0.93%). (B) Confocal imaging shows specific antibody binding for protoplasts expressing human claudin-1. The protoplasts were immuno-flourescently labelled with anti-claudin-1 or a relevant isotype control. Settings were optimized for each fluorescent protein to obtain the highest signal to noise ratio, while controlling for cross talk. Relevant isotype control antibodies did not bind.

Conformation-dependent antibodies that recognize native claudin-1 and, as a control, CD81 [21] were used as tools to probe the conformation of the yeast-expressed protein. In order to evaluate antibody binding by flow cytometry and confocal imaging, protoplasts were generated for each *P. pastoris* strain by enzymatically digesting the cell wall. Antibodies bound specifically to claudin-1 (33.6%) (**Fig. 2A, B**). In contrast, X33 control protoplasts (lacking heterologous protein expression) did not show significant antibody binding (**Fig. 2A, B**). These data provide evidence for correctly folded, antigenic claudin-1 in the yeast membrane.

### Biophysical characterization of recombinant human claudin-1

**Figure 3:**
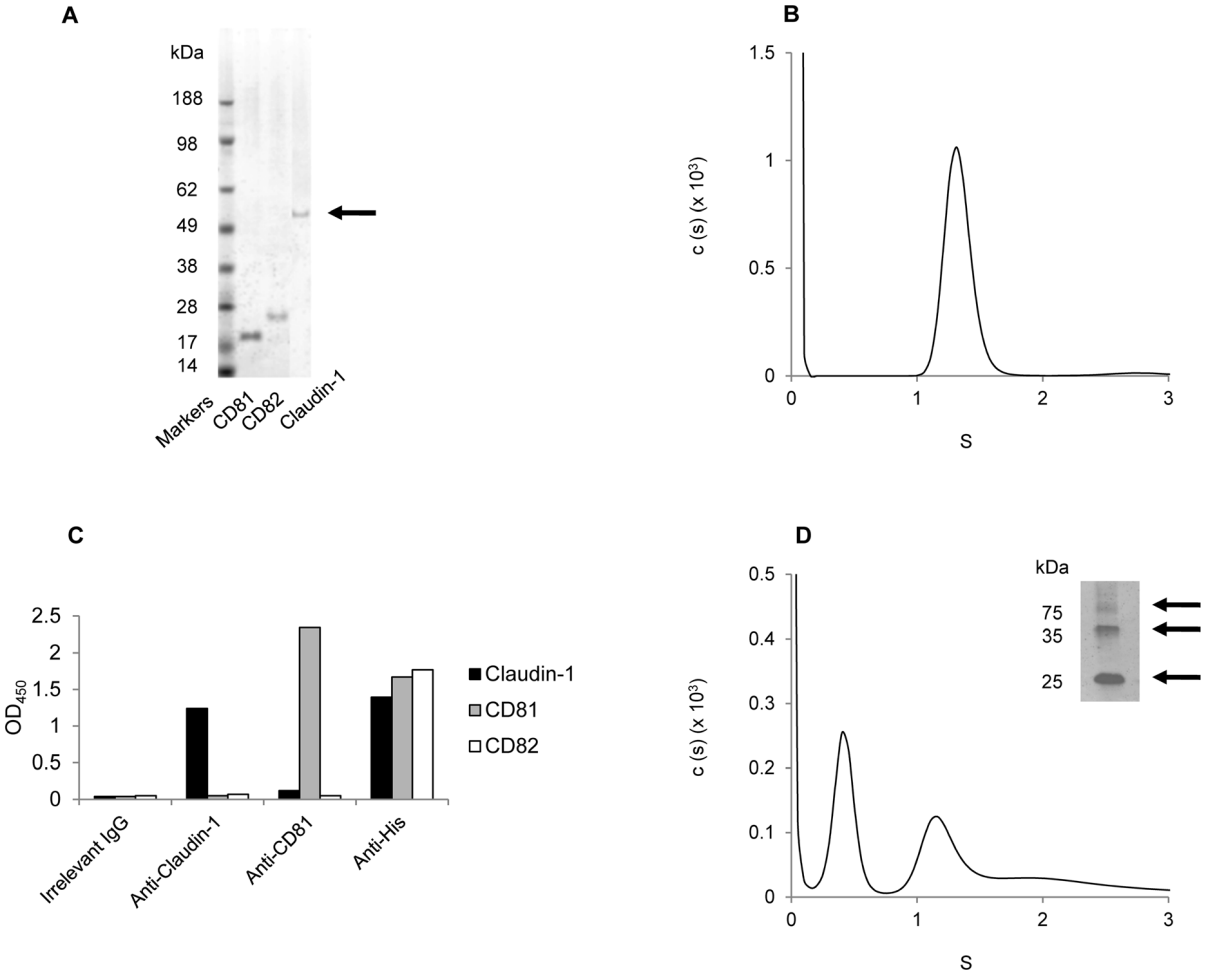
Biophysical analysis of purified claudin-1. (A) Coomassie-stained SDS-PAGE gel showing CD81 (lane 1 containing 2.4 µg protein), a control 4TM protein (CD82; lane 2; 1.5 µg protein) and claudin-1 (lane 3; 1.2 µg protein) solubilised in βOG and eluted from a nickel affinity column. Claudin-1 (highlighted with an arrow) runs as an apparent dimer. Molecular size markers are indicated. (B) Analytical ultracentrifugation trace for claudin-1 in βOG micelles collected on a Proteome Lab XL-I instrument. (C) ELISA data based on OD_450_ readings showing antibody reactivity (n = 2; error bars are the standard deviation) for βOG-extracted and purified claudin-1, CD81 and CD82. (D) Analytical ultracentrifugation trace for claudin-1 in profoldin-8 micelles collected on a Proteome Lab XL-I instrument. Data were analyzed using the continuous distribution, c(s), model, which calculates the distribution of sedimenting species taking into account their diffusion. Shown are the mass distributions of particles within the samples, c(s), as a function of the sedimentation co-efficient (S; measured in units of Svedberg, with 1 S = 10^−13^ s). Inset is a silver-stained SDS-PAGE gel showing claudin-1 solubilized in profoldin-8 and eluted from a nickel affinity column. Monomers and oligomers are highlighted with arrows.

Claudin-1 (23.7 kDa), CD81 (26.7 kDa) and the related tetraspanin CD82 (which is not a component of the HCV receptor complex; 30.6 kDa) were extracted from yeast membranes with βOG (**Fig. 3A**), a preferred detergent for biophysical studies [20], [22]. The three proteins were purified by nickel-affinity chromatography and size exclusion chromatography. Claudin-1 appeared as a dimer by reducing SDS-PAGE (**Fig. 3A**), whereas CD81 [20] and CD82 ran as monomers. AUC demonstrated claudin-1 was in a monodispersed oligomeric state (**Fig. 3B**). Antigenic characterization of purified, βOG-extracted claudin-1 and CD81 demonstrated reactivity with antibodies specific for the proteins and the hexahistidine tag (**Fig. 3C**).

Since claudin-1 formed higher molecular weight oligomeric structures in mammalian cell membranes (**Fig. 1B**), we screened further detergents in an attempt to isolate different oligomeric states from yeast membranes. Protein solubility was assessed in a range of micelles containing short-chain lipids and detergents with various acyl chains, head groups and amphipathic polymers including: n-dodecyl-β-d-maltopyranoside (DDM); foscholine-10; n-dodecylphosphocholine (DPC); n-dodecylphosphocholine-cholesterolhemisuccinate (DPC/CHS); 4-cyclohexyl-1-butylphosphocholine (cyclofos-4); 2,6-dimethyl-4-heptylphosphocholine; *N*,*N*-dimethyldodecan-1-amine oxide (LDAO); pentaethyleneglycol-n-octylether (C8E5); docosaethyleneglycol-monohexadecylether (Brij-58) and the profoldin membrane protein extraction solutions 1–12. Profoldin-8, a proprietary detergent formulation, most efficiently extracted oligomeric claudin-1 from yeast membranes. The circular dichroism (CD) spectrum of this material was consistent with profoldin-8-solubilized claudin-1 being folded in a predominantly α-helical state, with signature peaks at 208 nm and 222 nm. The corresponding AUC trace revealed two species (**Fig. 3D**), which could not be isolated by size exclusion chromatography. These data are consistent with the oligomeric states being in a dynamic equilibrium.

### Biological characterization of recombinant human claudin-1

**Figure 4:**
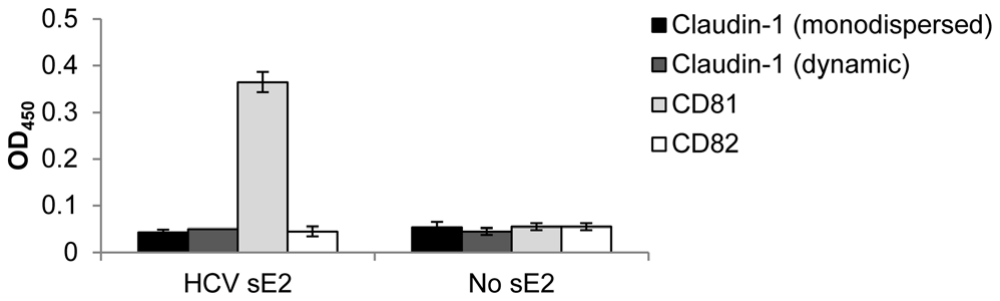
Purified claudin-1 does not bind HCV E2. ELISA data based on OD_450_ readings (n = 3; error bars are the standard deviation) showing reactivity with HCV E2. Claudin-1 in both βOG micelles (monodispersed) and profoldin-8 micelles (a dynamic mixture of oligomers) were analyzed and compared with CD81 (a positive control for HCV E2 binding) and CD82 (a negative control for HCV E2 binding). A control GNA lectin gave an OD_450_ signal of 3.29±0.04 in the presence of HCV E2, and 0.04±0.01 in its absence.

**Figure 5:**
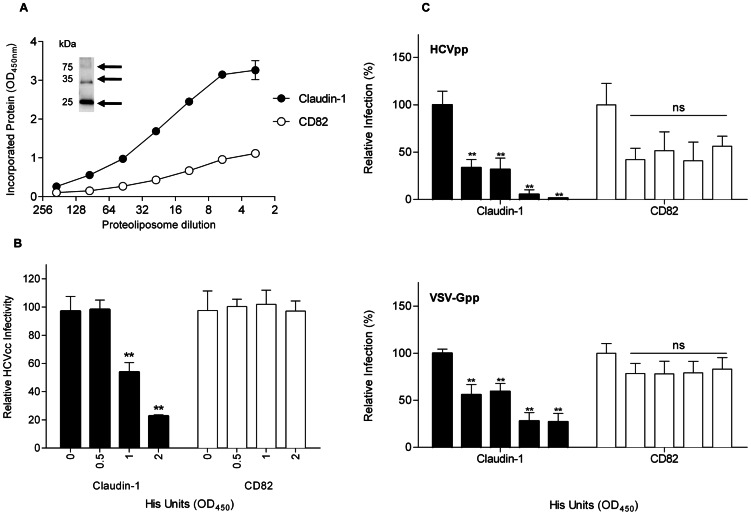
Biological analysis of claudin-1 in proteoliposomes. (A) Proteoliposome preparations containing claudin-1 or a control protein (CD82) were diluted and evaluated for anti-His reactivity by ELISA; data are represented as optical density (OD) at 450 nm. Inset is an immunoblot of foscholine-10 extracted claudin-1 stained with an anti-hexahistine tag antibody. Monomers and oligomers are highlighted with arrows. Proteoliposome preparations were normalized for His OD units and evaluated for their effect on (B) HCV as well as (C) HCVpp and VSV-Gpp infectivity. Data are expressed relative to untreated control virus infection.

To investigate whether purified claudin-1 can associate with HCV glycoproteins, monodispersed (βOG-extracted and purified) and oligomeric (profoldin-8-extracted and purified) forms were assessed for their ability to bind HCV sE2. Neither form of claudin-1 bound the virus glycoprotein (**Fig. 4**). In contrast, CD81 bound HCV sE2 as previously reported (**Fig. 4**; [20]). These data suggest that claudin-1 does not interact directly with HCV-encoded glycoprotein, as previously observed in human hepatocyte-derived cell lines [23]. For subsequent experiments, a replacement for the detergent profoldin-8 was sought on account of its proprietary composition: foscholine-10 also extracted oligomeric claudin-1 with the same properties as profoldin-8 (**Fig. 5A**), whilst being chemically defined.

Surfactants, such as the detergents used in our study, are not compatible with mammalian cell viability. To study the biological activity of claudin-1 *in vivo*, the purified protein and tetraspanin CD82 were therefore reconstituted into proteoliposomes [24]. We assessed protein incorporation by measuring anti-claudin-1, anti-CD82 or anti-His reactivity by Western blotting and ELISA (**Fig. 5A**). Since the hexahistidine tag is located at the carboxy-terminus of both proteins, we compared antibody reactivity to native and detergent-solubilized proteoliposome preparations to assess protein orientation. We routinely observed an increase in anti-His reactivity with detergent solubilized proteoliposomes compared with untreated ones (data not shown) suggesting that the proteins are incorporated in both orientations, as might be anticipated.

Proteoliposome preparations were normalized for equivalent protein incorporation (as determined by His OD_450_ units) prior to evaluating their effect on HCV infection. Claudin-1-containing proteoliposomes inhibited HCV strain J6/JFH infection of Huh-7.5 hepatoma cells in a dose-dependent manner, whereas CD82-containing control proteoliposomes had no effect (**Fig. 5B**). To determine whether claudin-1-containing proteoliposomes inhibit HCV infection at the level of particle entry, their effect on HCVpp and control VSV-Gpp was evaluated. Claudin-1-containing proteoliposomes inhibited HCVpp and VSV-Gpp infection (**Fig. 5C**). Control proteoliposomes (containing CD82) had no effect on HCVpp or VSV-Gpp infection (**Fig. 5C**). These data suggest that the presence of claudin-1 in proteoliposomes alters pseudoparticle uptake *per se* and that this modulation is not specific to HCV entry.

### Claudin-1 oligomers can associate with CD81 *in vitro*

In the absence of a tractable assay to study the biological function of claudin-1, we measured its ability to interact with CD81 *in vitro* in the absence of host cell proteins, lipids or other cellular components. *In vitro* mixing was first performed with βOG-solubilized claudin-1 since this preparation had been demonstrated to be monodispersed (**Fig. 3B**). Following a 30 min incubation, 1∶1 and 1∶2 molar ratios of βOG-solubilized claudin-1 and CD81, claudin-1 and CD82 or CD81 and CD82 were analyzed by size exclusion chromatography. In all cases, there was no substantial change in the elution profile compared with pure proteins alone, suggesting that no complexes had formed. Potential interactions were explored further using oligomeric claudin-1, monodispersed CD81 and, as a control, CD82.

Since tetraspanins are found in cholesterol-enriched membrane microdomains, where they interact with each other [25] and other proteins [26], we designed *in vitro* mixing experiments to examine the association of purified claudin-1 and CD81, claudin-1 and CD82 or CD81 and CD82 in different molar ratios (9∶1, 2∶1, 1∶1, 1∶2 and 1∶9) in the presence or absence of CHEMS; an acidic cholesteryl ester that self-assembles into bilayers in alkaline and neutral aqueous media [27]. DLS was used to analyze the time-dependent intensity fluctuations of scattered light in each mixture of solubilized proteins (including any interactions or complexes formed).

**Figure 6:**
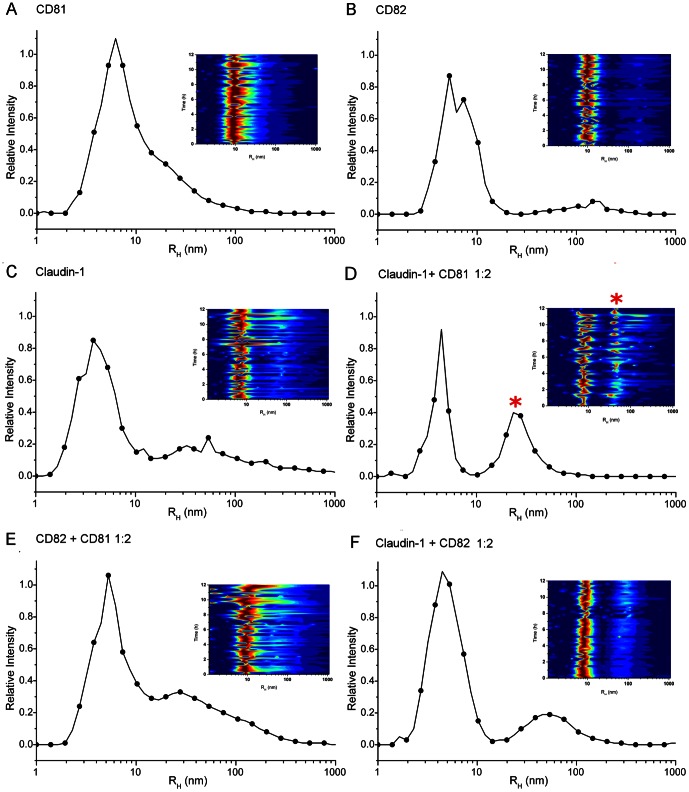
Oligomeric claudin-1 associates with CD81 at a specific molar ratio of 1∶2. A plot of relative intensity of differently-sized particles (R_H_ reported in nm, derived from DLS measurements at room temperature) of claudin-1, CD81 or a control protein (CD82), alone or as mixtures, solubilized in detergent micelles and in the presence of CHEMS. The data are the average of the first 10 measurements of the DLS experiment. Inset are DLS heat maps, where the colour spectrum indicates the amplitude of the signal for a given hydrodynamic radius (R_H_) value as a function of time; red is high- and blue is low amplitude. (A) CD81 in βOG. (B) CD82 in CD. (C) claudin-1 in foscholine-10; claudin-1 particles have a broad radial distribution consistent with a dynamic pool of oligomers. (D) claudin-1 mixed with CD81 in a 1∶2 molar ratio; a distinct 30 nm particle is highlighted with a red asterisk. (E) CD82 mixed with CD81 in a 1∶2 molar ratio; in addition to a dominant peak at 6 nm, some higher oligomers are present, which do not form a distinct peak. (F) claudin-1 mixed with CD82 in a 1∶2 molar ratio; in addition to a dominant peak at 6 nm, higher oligomers are present at >40 nm.

**Figure 7:**
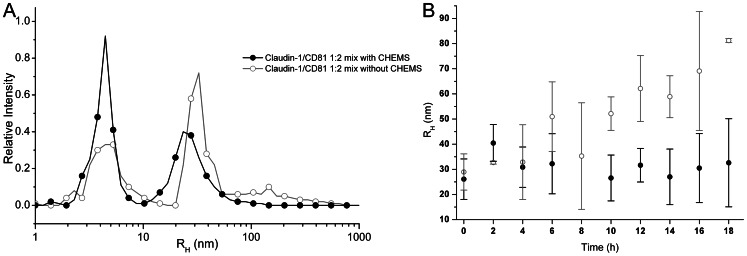
Claudin-1/CD81 complexes are stabilized by CHEMS. (A) A plot of relative intensity of differently-sized particles (R_H_ reported in nm) is shown for the claudin-1/CD81 1∶2 mix with (closed circles) and without (open circles) CHEMS. (B) The stable R_H_ = 30 nm particle forms within a few minutes of mixing and is stable in the presence of CHEMS for 18 h. In the absence of EC1, aggregation occurs after 4 h.

Within the DLS intensity fluctuation data, information is contained on the time scale of movement of all particles in solution and, importantly, their size distribution profile [28]. As previously described [29], hydrodynamic radii (R_H_) could be calculated for the particles contained in all mixtures analyzed (**Table 1**). To determine the diffusion coefficient, the CONTIN algorithm [30] was used to analyze the auto-correlation function (ACF). The Stoke-Einstein equation [31] was used to calculate R_H_ from these data. Two particle distributions were observed; one with R_H_<10 nm and one with R_H_≥10 nm. For claudin-1 solubilized with foscholine-10 in the presence or absence of CHEMS, the mean particle hydrodynamic radius of the smaller particle was 4.0 nm (**Fig. 6C** **;** **Table 1**). This corresponds to a molecular weight of approximately 77 kDa, consistent with a dimer; the molecular mass of claudin-1 is 23 kDa, while that of a foscholine-10 micelle, as determined by DLS, is 28 kDa [32]. The larger particle was defined by a peak which was broader than that for βOG-solubilized CD81 or βOG-solubilized CD82 (**Fig. 6A, B**), consistent with purified claudin-1 being composed of a dynamic mixture of oligomeric states (**Fig. 3D** **;** **Table 1**). Peak broadening in excess of 100 nm (**Fig. 6C**) was even more pronounced after 6 h of measurement. For all claudin-1∶CD81 ratios, except for claudin-1∶CD81 1∶2, the distributions of particle sizes were similar to the one observed for a non-interacting mixture such as CD81/CD82 (**Fig. 6E**) or claudin-1/CD82 (**Fig. 6F**). For claudin-1∶CD81 1∶2, the distribution of particle radii in solution was different: both with and without CHEMS, a defined oligomeric species at ∼30 nm appeared within 1 h of mixing the proteins (**Fig. 6D** **,** **Fig. 7** **;** **Table 1**), which was not observed for any other combination or ratio of 4TM proteins. The relative abundance of the 30 nm complex also increased, becoming dominant over 12 h (**Fig. 6D** **,** **7A**). This suggests that complex formation between claudin-1 and CD81 was only possible *in vitro* at a specific molar ratio of 1 claudin-1∶ 2 CD81 and that the complex was stabilized by the presence of CHEMS (**Fig. 7B**). These data provide evidence that dynamic pools of claudin-1 associate with CD81 and that the receptor complexes are stabilised in cholesteryl-enriched membranes. This occurs *in vitro* in the absence of other HCV receptor or cellular components.

**Table 1:** Distribution of particle hydrodynamic radii (R_H_) derived from DLS measurements of purified recombinant claudin-1 and CD81.

Protein	Particle radius (R_H_; nm)				Predicted oligomeric state
	− CHEMS		+ CHEMS		
	<10 nm	>10 nm	<10 nm	>10 nm	
Claudin-1 in foscholine-10	4.0 (2.5; 0.6)	40 (40; 0.3)	4.0 (1.3; 0.8)	40 (20; 0.2)	Dimer (4 nm) as well as higher oligomers/aggregates (peak at 40 nm)
CD81 in βOG	6.0 (3.5; 1.0)	–	6.5 (1.0; 1.0)	25 (6; 0.3)	Dimer (6 nm) plus, in the presence of CHEMS, a distinct peak at 25 nm
Claudin-1∶ CD81 (1∶1)	4.6 (2.0; 1.0)	45 (40; 0.2)	5.0 (1.6; 1.0)	48 (23; 0.2)	Dimer (4–5 nm) as well as higher oligomers/aggregates (broad peak at >40 nm)
Claudin-1∶ CD81 (1∶2)	4.2 (0.9; 0.9)	28 (9.0; 0.4)	4.0 (1.6; 0.3)	33 (9.0; 0.7)	Dimer (4 nm) as well as a distinct oligomer (30 nm) which is dominant in the presence of CHEMS; predicted to be hexameric
Claudin-1∶ CD81 (2∶1)	4.3 (1.7; 0.8)	42 (10; 0.2)	5.0 (1.0; 0.9)	68 (30; 0.2)	Dimer (4–5 nm) as well as higher oligomers/aggregates (broad peak at >40 nm)
Claudin-1∶ CD81 (1∶9)	5.2 (1.4; 0.9)	48 (48; 0.3)	6.5 (2.6; 0.9)	70 (85; 0.2)	Dimer (>5 nm) as well as higher oligomers/aggregates (broad peak at >40 nm)
Claudin-1∶ CD81 (9∶1)	4.5 (2.5; 0.8)	75 (30; 0.2)	4.0 (0.5; 1.0)	41 (10; 0.2)	Dimer (>4 nm) as well as higher oligomers/aggregates (peak at 40 nm similar to claudin-1 alone)

Values are reported after 12 h for particles with hydrodynamic radii <10 nm or >10 nm, as defined by DLS peaks. Peak width at half height (nm) and relative peak amplitude are reported in parentheses, respectively. Amplitude is relative to that of the corresponding CD81 peak in the absence of CHEMS. The radius of a foscholine-10 micelle in solution, calculated from DLS measurements, is 2.5±0.6 nm [32]. For βOG-micelles, the value is 2.3–3.1 nm [54].

## Discussion

Since no high resolution crystal structure of any full-length HCV receptor component has been published, new approaches to facilitate their biophysical characterization are required. In this study we demonstrate that recombinant, correctly-folded, full-length claudin-1 can be produced in yeast membranes, that it can be extracted in different oligomeric forms that do not bind HCV sE2 and that a dynamic preparation can complex with CD81 *in vitro* at a defined molar ratio of 1∶2. This event does not require any other cellular component(s) and complexes are stabilized in cholesteryl-enriched membranes.

Previous biophysical studies (using AUC and light scattering) suggest that recombinant claudin-4 is in a dynamic equilibrium of hexamers and other oligomers [33]. A hexameric structure aligns with the low resolution structural analysis of the tetraspanin uroplakins (UPs) Ia and Ib, which have been shown to comprise a hexamer of heterodimers that form a crystalline 2D array *in vivo* of approximately 16 nm particles (R_H_≈8 nm) [34]. This dynamic behavior is consistent with tight junction strand breakage and reformation, which is important for barrier function. Our *in vitro* data for purified recombinant claudin-1 in detergent micelles suggest that dynamic populations of claudin-1 are capable of rapidly forming complexes with CD81 that are stabilized in a cholesteryl-enriched environment. Cholesterol is crucial for HCV entry [35] and a 3D model of CD81 reveals an aromatic amino acid cluster in the membrane that is typical of two cholesterol binding sites [36]. Notably, tetraspanins are stabilized by the cholesterol enrichment of microdomains [37] and mammalian cells depleted of cholesterol show lower claudin-1/CD81 interaction by FRET that is restored by cholesterol addition [3].

**Figure 8:**
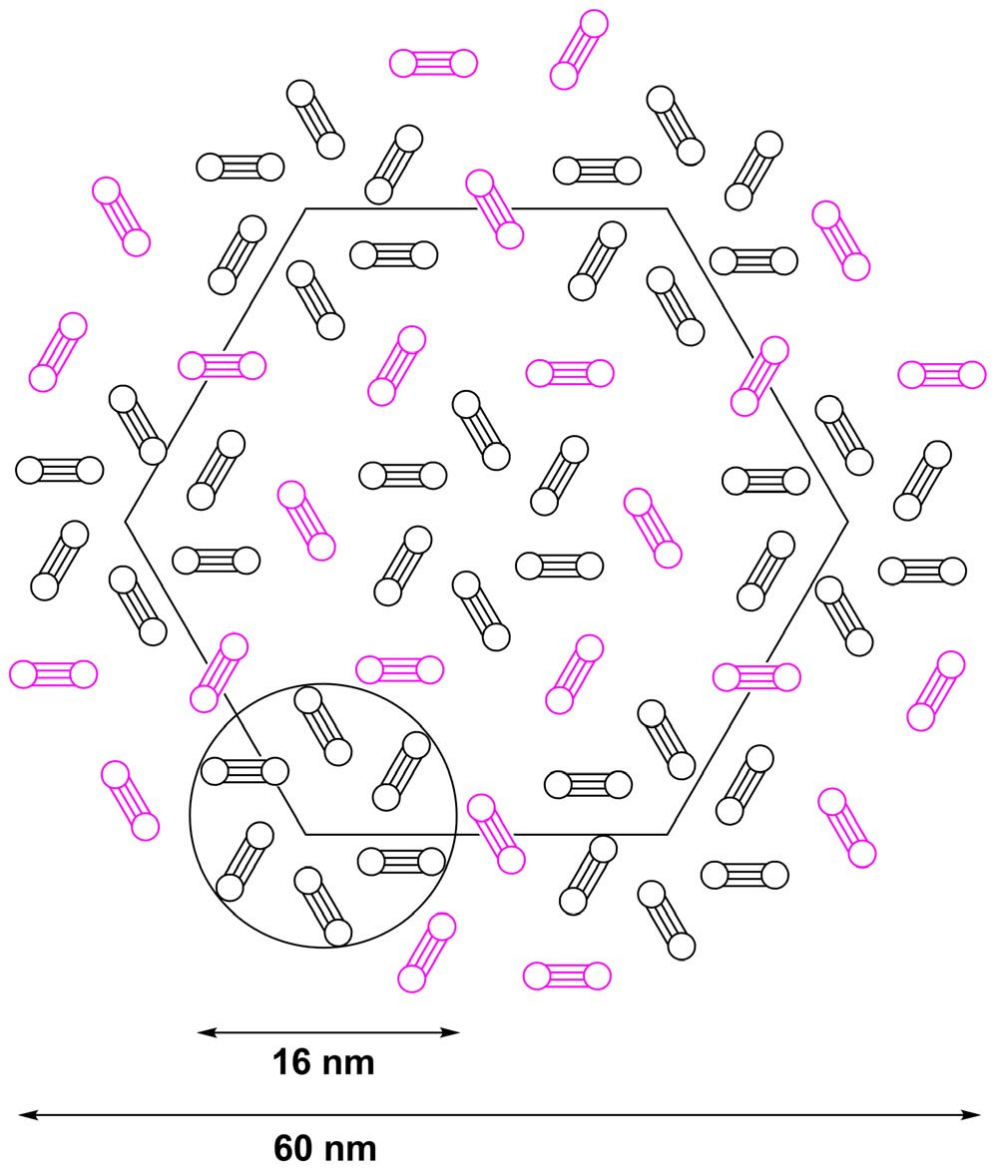
Proposed assembly of claudin-1/CD81 complexes (R_H_ = 30 nm). A cartoon assembled in ChemDraw illustrating seven uroplakin-like CD81 complexes (black), each containing six homodimers, arranged with 24 claudin-1 homodimers (red). The resulting molar ratio of 24∶42 claudin-1∶CD81 is close that observed experimentally (Table 1), and generates a R_H_ = 30 nm particle. The model displayed is the most symmetric of those generated.

We previously reported a 1∶1 stoichiometry of the claudin-1/CD81 complex in mammalian cells using FRET. To rationalise this observation with our DLS data (**Table 1**), the tetraspanin uroplakin model [34] and evidence that the fundamental structural unit of CD81 [19] and claudins [38] is a dimer, we propose the schematic representation of a possible claudin-1/CD81 complex (**Fig. 8**). This scheme is based on stoichiometry derived from low resolution cryo-electron crystallography data [34] and is therefore speculative: seven uroplakin-like CD81 complexes (black), each containing six homodimers, are arranged with 24 claudin-1 homodimers (red) to generate a symmetrical particle, which fits with our data (R_H_ = 30 nm; **Table 1**). The resulting molar ratio of 24∶42 claudin-1∶CD81 is close to the observed 1∶2 ratio observed in our DLS experiments (**Table 1**). At the center of the complex, 6 claudin-1 dimers are packed near 6 CD81 dimers, an arrangement that would account for our previous FRET data [10] for dimers packed within approximately 10 nm of each other; at larger distances the FRET signal decreases sharply as it has an inverse sixth power dependence on distance. This hypothetical complex is consistent with a recent study demonstrating that in mouse liver tissue, claudin-2 can be isolated as a component of a high molecular weight protein complex with claudin-1 and occludin [38] and that claudin-2 has been reported to dimerize via transmembrane interactions [38].

Single particle tracking studies demonstrate that CD81 is highly mobile in the membrane [39], [40] and claudin-1 expression reduces CD81 mobility (Harris, unpublished data). The data presented here support a model whereby CD81 diffuses through the membrane and is able to complex with claudin-1 on account of its ability to exist in a dynamic equilibrium of homo-oligomeric (**Fig. 3D**) and hetero-oligomeric (**Table 1** **,** **Fig. 7** **,** **Fig. 8**) forms. At a molar ratio of claudin-1∶CD81 of 1∶2, a complex is formed further consistent with reports of other high molecular weight protein complexes containing claudin-1 [38].

To analyze the biological function of claudin-1 (and its complexes), we generated proteoliposomes; recent advances in the use of polymers to isolate membrane proteins and their complexes in a biocompatible format may also offer a future solution for studies of biological function [41]. Unexpectedly, we found that the presence of claudin-1 in proteoliposomes altered pseudoparticle uptake into hepatoma cells and that this modulation was not specific to HCV (**Fig. 5**). Polyunsaturated ER-targeting liposomes (PERLs) have also been demonstrated to be anti-viral, affecting both the secretion and infectivity of HIV, hepatitis B and hepatitis C viruses [42]. Cholesterol levels in the plasma membrane of the host cell and the membrane of the secreted virus were disrupted by PERLs [42]. Claudin-1-containing proteoliposomes, like PERLs, also appear to inhibit HCV entry; since claudin-1 is required in the late stages of HCV cell entry [6], it is possible that the internalization of claudin-1-containing proteoliposomes causes this inhibition, although this remains to be determined [35].

Both structural and functional analyses of recombinant claudin-1 with CD81 and HCV are now required to identify the critical amino acids defining HCV receptor form and function [6]. The data presented here pave the way for the structural characterization of wild-type and mutant forms of claudin-1, alone and in complex with CD81, which may ultimately enable structure-aided design of new therapeutic agents targeted at viral entry.

## Materials and Methods

### Production of recombinant claudin-1, CD81 and CD82 and protoplast formation

Recombinant, full-length 4TM proteins, with a hexahistidine tag at the carboxy terminus, were produced in *P. pastoris* X33 cells under the control of the *AOX1* promoter using the pPICZB vector (Invitrogen) [43], [44], as previously described for CD81 [20], [45]. The full-length CD81 protein used in this study was previously produced in a form where six putative palmitoylation sites were mutated to alanine [20]; this mutant form of CD81 binds the HCV envelope [20], [46] and permits viral entry [46]. Full-length claudin-1 and CD82 were wild-type. To generate protoplasts, a mid-logarithmic phase aliquot of *P. pastoris* X33 cells producing the relevant 4TM protein was re-suspended in 50 mM KH_2_PO_4_, 40 mM β-mercaptoethanol (adjusted to pH 7.2 with KOH) and incubated for 30 min at 30°C, 150 rpm. The cell suspension was diluted 1∶1 with the same 50 mM phosphate buffer including 2.4 M sorbitol. Zymolyase 20T was added to a final concentration of 1 mg mL^−1^ and the reaction was incubated for 90 min at 30°C, 150 rpm. After harvesting the protoplasts (500× g, 5 min) and washing them once, they were resuspended in a stabilizing buffer (250 mM KCl, 10 mM CaCl_2_, 5 mM MgCl_2_, 5 mM MES adjusted to pH 7.2 with Tris-base) supplemented with 1% glucose.

### Fluorescent activated cell sorting

Protoplasts expressing recombinant claudin-1 or no heterologous protein were blocked in phosphate buffered saline (PBS) using 1% bovine serum albumin for 20 min at room temperature. Protoplasts were washed in PBS and then incubated with primary antibodies; anti-CD81 (2s131; [21]), anti-claudin-1 (R&D systems), anti-His (Clontech) or a control isotype monoclonal antibody for 1 h at room temperature. After washing, secondary antibody Alexa Fluor 488 goat anti-mouse IgG (H+L) or Alexa Fluor 633 goat anti-rat IgG (H+L) (Invitrogen) was added for 1 h to detect the bound primary antibody. Washing subsequently removed unbound antibody and samples were analysed using fluorescent activated cell sorting.

### Laser scanning confocal microscopy

Protoplasts expressing either human claudin-1 or no heterologous human proteins (X33) were seeded onto glass coverslips. The protoplasts were immuno-flourescently labelled for human claudin-1 (anti-claudin-1, R&D systems) or a non-specific IgG (anti- mouse IgG). Confocal optical sectioning (45 Z sections, 0.42 µm) was performed on an upright Zeiss 780 laser scanning confocal microscope using a 100×1.4NA objective. Microscope settings were optimized for each fluorescent protein to obtain the highest signal to noise ratio, while controlling for cross talk. 3D reconstructions of the 16 bit optical sections were performed using the Carl Zeiss ZEN software.

### Western blotting

Yeast membranes were prepared from 20 g wet cells suspended in 40 mL sterile ice-cold breaking buffer (50 mM sodium phosphate buffer, 2 mM EDTA, 100 mM NaCl, 5% glycerol (w/v), pH 7.4) and protease inhibitors (cocktail set IV, Calbiochem). Cells were passed through an Emulsiflex-C3 cell disrupter until >90% breakage was observed under a light microscope. Unbroken cells and cell debris were removed by centrifugation (10,000× g, 30 min, 4°C) and the supernatant was collected and ultracentrifuged (100,000× g, 90 min, 4°C) to isolate the total membrane fraction. The yeast membrane pellet was suspended in sterile ice-cold buffer (20 mM HEPES, 50 mM NaCl, 10% glycerol, pH 7) using a glass homogenizer. Membrane fractions were kept on ice if used for immediate analysis or snap-frozen in liquid nitrogen and stored at −80°C. For reducing SDS-PAGE total membrane samples were heated at 98°C for 10 min in sample buffer (50% distilled water, 12.5% 0.5M Tris-HCl (pH 6.8), 10% glycerol, 2% SDS, 5% β-mercaptoethanol and 0.001% bromophenol blue). Non-reducing SDS-PAGE samples were prepared using the same buffer without the addition of β-mercaptoethanol. Samples were separated using a 12% Tris-HCl gel and transferred to a nitrocellulose membrane before immunoblotting with a primary monoclonal antibody (anti-His_6_, Clontech) followed by an anti-mouse IgG HRP-conjugated secondary antibody (Sigma). EZ-ECL chemiluminescence (Geneflow) was used to detect protein bands.

Huh-7.5 hepatoma cells (the kind gift of Charles Rice, The Rockefeller University [47]; seeded the preceding day at 1.5×10^4^ cm^−2^) were harvested in lysis buffer (PBS, 1% Triton-X100, 0.1% sodium deoxycholate, 0.1% SDS) containing protease inhibitors (Complete, Roche, UK). Cell lysates were clarified by centrifugation (20,000× g, 30 min) and the protein concentration determined using Protein Assay Reagent (Pierce) according to manufacturer's instructions. Quantified protein lysates were separated by 12% SDS-PAGE and transferred to PVDF membranes (Sigma, UK) for incubation with anti-claudin-1 (MH25, Invitrogen). Secondary antibodies, horseradish peroxidase-conjugated donkey anti-rabbit, were detected by enhanced chemiluminescence (Geneflow, UK).

### Quantitative ELISA

Purified tetraspanin proteins or enriched membranes were allowed to bind Immulon II ELISA plates (Nunc) at 1 µg mL^−1^ for 4 h at 37°C. Unbound protein was removed by washing with PBS, the plates were blocked with 5% bovine serum albumin-PBS and bound protein detected with anti-receptor antibodies and anti-species, immunoglobulin-horseradish peroxidase conjugate (Jackson Laboratories) and tetramethylbenzidene (BioFX Laboratories). In parallel, tetraspanin proteins were evaluated for their ability to bind recombinant HCV E2. HCV E2_661_ strain H77 was used at 1 µg mL^−1^ by incubating at 37°C for 2 h and bound antigen detected with anti-E2 3/11, as previously reported [48]. Absorbance values were measured at 450 nm on a Fusion Plate Reader (Perkin-Elmer).

### Purification of recombinant claudin-1, CD81 and CD82

CD81 and CD82 were extracted from yeast membranes using 3% βOG (Anatrace) at 15°C for 1 h. Claudin-1 was solubilised using 3% βOG [20], [45], 3% profoldin-8 or 3% foscholine-10 (Anatrace) at 15°C for 16 h. Solubilized proteins were purified using HisTrap HP sepharose (GE Healthcare) with elution at 500 mM imidazole [20], [45]. Eluted fractions were concentrated at 10°C using a VivaSpin20 column, with a 10 kDa cut-off, prior to loading onto a HiLoad Superdex S200 gel filtration column, also equilibrated at 10°C. Proteins were eluted in 10 mM MOPS pH 8, 1% βOG at a flow rate of 0.2 mL min^−1^. Purity and yield were assessed using Coomassie blue staining of SDS-PAGE gels. Gels were also silver stained (SilverStainPlus, BioRad) and the identity of the 4TM proteins was confirmed by immunoblot using specific antibodies for CD81 [49] and claudin-1 (Zymed; 51-9000). The yield of purified CD81 and CD82 from 2 g wet weight of membranes, solubilized in 20 mL 3% detergent solution, was approximately 2 mg, whilst the corresponding yield of claudin-1 was approximately 0.3 mg. Multiple protein preparations were generated during this study to enable subsequent analyses.

### Biophysical analysis of purified 4TM proteins

A Beckman XLI analytical ultracentrifuge using an 8 cell 50Ti rotor was used for the AUC studies according to [20]. A total of 100 measurements were taken throughout each 8 h run. Data from each experiment were analyzed using the continuous c(s) distribution model implemented within SEDFIT94. Parameters for the partial specific volume of the protein, buffer viscosity and density were calculated using SEDNTERP [20]. CD spectra were recorded using a JASCO J-810 nitrogen-flushed spectropolarimeter, as previously described [20].

### Biological analysis of claudin-1

Proteoliposomes were generated from purified, recombinant claudin-1 (profoldin-8-extracted and purified) and CD82 (βOG-extracted and purified) as previously described [24] using cholesterol∶sphingomyelin∶1,2-dioleoyl-sn-glycero-3-phosphocholine at a molar ratio of 24∶37∶37. Lipids were mixed with protein at a molar ratio of 5,000∶1 at room temperature in the presence of 1.6% β-OG in 1 mL 25 mM HEPES pH 7.5, 150 mM NaCl. Proteoliposomes were recovered by flotation in an OptiPrep gradient. Dialyzed and pooled proteoliposomes were used for subsequent viral assays. SDS-PAGE and silver staining confirmed they contained protein; the lipid composition of the proteoliposomes was 30% cholesterol, 55% sphingomyelin, 15% 1,2-dioleoyl-sn-glycero-3-phosphocholine. For viral infection assays, HCV J6/JFH was generated as previously described [50]. Briefly RNA was transcribed *in vitro* from full-length genomes (RiboMax T7 kit, Promega) and electroporated into Huh-7.5 cells. Viral stocks were generated by two sequential passages through Huh-7.5 cells. Supernatants were collected, pooled, and stored at −80°C. Infected cells were detected by methanol fixation and staining for HCV encoded NS5A using monoclonal antibody, 9E10 (a gift of Charles Rice and Tim Tellinghuisen Rockefeller University, USA); bound antibody was detected with anti-mouse IgG Alexa-488 and quantified by enumerating NS5A expressing cells. Pseudoviruses expressing a luciferase reporter were generated as previously described [51]. Briefly, 293T cells were transfected with a 1∶1 ratio of plasmids encoding HIV provirus expressing luciferase and HCV strain H77 E1E2 envelope glycoproteins (HCVpp), vesicular stomatitis virus G glycoprotein (VSV-Gpp) or empty vector (Env-pp). Supernatants were harvested 48 h post transfection. Infection was quantified by measuring luciferase activity (relative lights units, RLU) and specific infectivity determined by subtracting the mean Env-pp signal from the HCVpp, or VSV-Gpp values. HCVcc or HCVpp were allowed to infect Huh-7.5 cells in the presence or absence of proteoliposome preparations for 6 h and unbound virus removed by washing and the cells fed with 3% FBS/DMEM. Infections were allowed to proceed for 72 h and enumerated by counting NS5A expressing cells or luciferase activity, respectively.

### Analysis of complex formation by size exclusion chromatography and DLS

Initial analysis of complex formation was done by size exclusion chromatography (Superdex S200 10/300 GL; column volume 24 mL) on solubilized proteins alone and after incubation together. Subsequently, DLS measurements were used to assess whether complexes had formed. Prior to mixing, to avoid disturbances to the DLS measurements, aggregates and dust particles were removed from the purified protein solutions (0.3–0.4 mg mL^−1^) by filtration through a 0.1 mm filter, followed by clarification at 14,000× g. Proteins were then transferred to an MRC crystallization plate (96-well SBS format, Swissci AG, Switzerland) at 1 µL/well. Five different molar ratios of claudin-1/CD81, claudin-1/CD82 and claudin-1/claudin-1 (9∶1; 2∶1; 1∶1; 1∶2; 1∶9) were analyzed with and without the addition of 0.1 µL CHEMS (2.2 mg mL^−1^ in 20 mM MOPS, pH 8, 1% beta-OG) to the drop. As controls, claudin-1, CD81 and CD82 were analyzed alone, with and without addition of 0.1 µL CHEMS (2.2 mg/mL in 20 mM MOPS, pH 8, 1% βOG) to the drop, as well as the same buffer without protein. The reservoirs were filled with 35 µL protein buffer (20 mM MOPS, pH 8, 1% beta-OG) and the plate was sealed with SmartSeal (Greiner Bio-One GmbH, Germany) to avoid evaporation during the measurements. DLS measurements within these droplets were carried out at 20°C using a *Spectro* Light 500 instrument (XtalConcepts GmbH, Germany), a plate reader for UV/visible-imaging and *in situ* DLS [52]. Data were analyzed using *Spectro* (Nabitec GmbH, Germany). *Spectro* interprets the autocorrelation function (ACF) [53] using the CONTIN-algorithm [30] to obtain the distribution of particle radii. To obtain data on the time-dependent change of the hydrodynamic radius (R_H_) distribution of the respective protein mixtures, DLS measurements were recorded for 23–36 h in all wells. From each well a series of 130 measurements at 30 s per measurement was recorded, with an interval of approximately 10 min between each measurement.
